# Lectures on the Action of Medicines

**Published:** 1898-06

**Authors:** 


**Lectures on the Action of Medicines.** By T. LAUDER BRUNTON, M.D., F.R.S. Pp. xv., 673. London: Macmillan and Co., Ltd. 1897.

Of the nature and description of the contents of this book we shall not permit ourselves to say more than that it is a reprint of a shorthand report of Dr. Lauder Brunton's extempore lectures on pharmacology and therapeutics to the students at St. Bartholomew's Hospital. We refrain designedly from giving any further information about the book, because it is one which every medical man should possess and use for instruction, relaxation, and enjoyment. For variety and freshness of interest, for stirring incident, for pointed anecdote, for entertainment and for general mental and moral improvement (as well as for the abundant technical information so ably afforded) we have no hesitation in saying that this book far excels the modern novel, and should be the guide, philosopher, and friend of every practitioner. In *The Beth Book* we are told that after conversation of a certain description, when Beth retired to bed "she was like one who has been bathed and perfumed after the defilements of a long journey." The perusal of Dr. Brunton's book after a long day's work will make the weary practitioner feel as if he also had been bathed and perfumed, without having previously wallowed in sexual mire.

